# A Semi-supervised Learning Method for Q-Matrix Specification Under the DINA and DINO Model With Independent Structure

**DOI:** 10.3389/fpsyg.2020.02120

**Published:** 2020-09-10

**Authors:** Wenyi Wang, Lihong Song, Shuliang Ding, Teng Wang, Peng Gao, Jian Xiong

**Affiliations:** ^1^School of Computer and Information Engineering, Jiangxi Normal University, Nanchang, China; ^2^Elementary Education College, Jiangxi Normal University, Nanchang, China

**Keywords:** cognitive diagnostic assessment, Q-matrix, the augment algorithm, the reachability matrix, the conjunctive model, the disjunctive model

## Abstract

Cognitive diagnosis assessment (CDA) can be regarded as a kind of formative assessments because it is intended to promote assessment for learning and modify instruction and learning in classrooms by providing the formative diagnostic information about students' cognitive strengths and weaknesses. CDA has two phases, like a statistical pattern recognition. The first phase is feature generation, followed by classification stage. A Q-matrix, which describes the relationship between items and latent skills, corresponds to the feature generation phase in statistical pattern recognition. Feature generation is of paramount importance in any pattern recognition task. In practice, the Q-matrix is difficult to specify correctly in cognitive diagnosis and misspecification of the Q-matrix can seriously affect the accuracy of the classification of examinees. Based on the fact that any columns of a reduced Q-matrix can be expressed by the columns of a reachability R matrix under the logical OR operation, a semi-supervised learning approach and an optimal design for examinee sampling were proposed for Q-matrix specification under the conjunctive and disjunctive model with independent structure. This method only required subject matter experts specifying a R matrix corresponding to a small part of test items for the independent structure in which the R matrix is an identity matrix. Simulation and real data analysis showed that the new method with the optimal design is promising in terms of correct recovery rates of q-entries.

## Introduction

In educational assessment, cognitive diagnostic assessment (CDA) that combines psychometrics and cognitive science has received increased attention recently (Leighton and Gierl, 2007; Tatsuoka, 2009; Rupp et al., 2010). This approach potentially provides useful diagnostic information regarding students' strengths and weaknesses, and can facilitate individualized learning (Chang, 2015). Cognitive diagnostic models (CDMs) often utilize a Q-matrix (Embretson, 1984; Tatsuoka, 1990, 1995, 2009). Tatsuoka (2009) pointed out that “Tatsuoka (1990) organized the underlying cognitive processing skills and knowledge that are required in answering test items correctly in a Q-matrix, in which the rows represent attributes and the columns represent items.” The entries of a Q-matrix are 1 or 0, denoted by *q*_*kj*_. If attribute *k* is involved in correctly answering item *j*, then *q*_*kj*_ = 1, and *q*_*kj*_ = 0 otherwise. The definition of Q-matrix in Tatsuoka (1990) is used in our study. Recently, one common representation of a Q-matrix is that in which the rows represent items and the columns represent attributes (Ma and de la Torre, 2020; Zhan et al., 2020). It should be noted that the representation of the Q-matrix that they used in the study differs from the traditional one.

Cognitive diagnostic assessment has two phases, like statistical pattern recognition and classification methodology. The first phase is feature generation, and then classification stage follows. The specification of Q-matrix corresponds to the feature extractor phase in statistical pattern recognition and classification problems. Feature generation is of paramount importance in any pattern recognition task. So, the Q-matrix plays a very important role in establishing the relation between latent attribute patterns and ideal/latent response patterns.

In practice, the Q-matrix is difficult to specify correctly in cognitive diagnostic assessment (Jang, 2009; DeCarlo, 2011) and misspecification of the Q-matrix can seriously affect the accuracy of both item parameter estimates and the classification of examinees (de la Torre, 2008; Rupp and Templin, 2008). Researchers have proposed several quantitative methods for deriving or refining Q-matrix. These methods can be classified into two categories (Xu and Desmarais, 2018): (a) the unsupervised method, including but not limited to the q-matrix method (Barnes, 2003, 2011), the non-negative matrix factorization technique (Desmarais, 2011; Desmarais et al., 2012; Desmarais and Naceur, 2013) or alternate least-square factorization method (Desmarais et al., 2014; Xu and Desmarais, 2016), the data-driven approach (Liu et al., 2012, 2013), and the exploratory factor analysis method (Barnes, 2003; Close, 2012; Wang et al., 2018b, 2020), and (b) the supervised method, including the sequential EM-based δ method (de la Torre, 2008) and its extension ς^2^ method (de la Torre and Chiu, 2016), the Bayesian approach (DeCarlo, 2012), the non-parametric Q-matrix refinement method (Chiu, 2013), the stepwise reduction algorithm (Hartz, 2002), the EM-based methods (Wang et al., 2018a), the residual-based or item fit statistic approach (Chen, 2017; Kang et al., 2018) and so on.

The unsupervised method is deriving a Q-matrix only from test data or item responses. The unsupervised method is very useful because there are many existing tests without specifying the Q-matrix but with test response data. However, it would be difficult to identify the number of latent skills and be slightly more difficult to understand results from real data. A study of Beheshti et al. (2012) found that the number of latent skills estimated from real data is not well-aligned with the assessment of experts.

The supervised method can incorporate the information of experts' Q-matrix and test response data to refine or validate the provisional Q-matrix. If the provisional Q-matrix is unknown for an existing test, the supervised methods cannot be used. Furthermore, this method often needs a high-quality provisional Q-matrix for a whole test. If the provisional Q-matrix is specified by subject matter experts but contains a large amount of misspecification, it will be difficult for the recovery of a high-quality Q-matrix through the supervised method, because the performance of the supervised method relies on the precision of classification of attribute patterns resulting from the provisional Q-matrix (de la Torre, 2008; Rupp and Templin, 2008).

Specifying a Q-matrix for a whole test by experts can be a time-consuming and fatigue process. The purpose of this study is to propose a semi-supervised method for Q-matrix specification in order to check whether only some of items needs to be identified by experts. The semi-supervised method falls between unsupervised and supervised methods.

## Model and Method

### Model

Let *K* be the number of attributes. Let *X*_*ij*_ be a binary random variable to denote the response of examinee *i* to item *j*, *i* = 1, 2, …, *N*, *j* = 1, 2, …, *J*. Let **α**_*i*_ be a column vector to denote an attribute mastery pattern or a knowledge state from the universal set of knowledge states. Moreover, Q-matrix that specifies the item-attribute relationship is a *K* × *J* matrix, in which entry *q*_*kj*_ = 1 if attribute *k* is required for answering item *j* correctly; otherwise, *q*_*kj*_ = 0.

The item response function for the deterministic inputs, noisy “and” gate (DINA) model (Haertel, 1989; Junker and Sijtsma, 2001; Chiu and Douglas, 2013) is as follows:

(1) $$\begin{array}{l} P_{j}\left(\alpha_{i}\right)=P\left(X_{ij}=1|\alpha_{i}\right)=g_{j}^{1-\eta_{ij}}{\left(1-s_{j}\right)}^{\eta_{ij}}, \end{array}$$

where a deterministic latent response $\eta_{ij}=\prod_{k=1}^{K}\alpha_{ki}^{q_{kj}}$ indicates whether or not examinee *i* possesses all of the attributes required by item *j*. A value of η_*ij*_ = 1 means that examinee *i* has mastered all of the attributes required by item *j*, and η_*ij*_ = 0 otherwise. The slip parameter *s*_*j*_ refers to the probability of an incorrect response to the item *j* when η_*ij*_ = 1, and the guessing parameter *g*_*j*_ refers to the probability of a correct response to item *j* when η_*ij*_ = 0. Let **B** = (η_*ij*_) be a deterministic latent response matrix for the DINA model.

The item response function for the deterministic inputs, noisy “or” gate (DINO) model (Templin and Henson, 2006; Chiu and Douglas, 2013) is as follows:

(2) $$\begin{array}{l} P_{j}\left(\alpha_{i}\right)=P\left(X_{ij}=1|\alpha_{i}\right)={\left(1-s_{j}\right)}^{w_{ij}}g_{j}^{1-w_{ij}}, \end{array}$$

where $w_{ij}=1-\prod_{k=1}^{K}{\left(1-\alpha_{ki}\right)}^{q_{kj}}$ is a deterministic latent response. As in the DINA model, *s*_*j*_ and *g*_*j*_ are the slip and guessing parameters of item *j*. The DINA and DINO model are conjunctive and disjunctive models (Maris, 1999), respectively. Let **W** = (*w*_*ij*_) be a deterministic latent response matrix for the DINO model.

#### A Semi-supervised Learning Approach for the Conjunctive Model

In the rule space method (Tatsuoka, 2009) or the attribute hierarchy method (Leighton et al., 2004), the adjacency matrix denoted by **A** represents the direct relationship among attributes. We denote the entry in row *k*_1_ and column *k*_2_ of **A** by *a*_*k*_1_*k*_2__. If a direct prerequisite relation exists from attribute *k*_1_ to attribute *k*_2_, then *a*_*k*_1_*k*_2__ = 1, and *a*_*k*_1_*k*_2__ = 0 otherwise. Let **R** denote a reachability matrix of order (*K, K*) to specify the direct and indirect relationships among attributes. The **R** matrix is given by **R** = (**A** + **I**)^*K*^ with respect to Boolean operations, where **I** is an identity matrix. The reduced Q matrix denoted by **Q**_**r**_ is obtained by removing the items (columns) that do not satisfy the specified relationships from the incidence Q matrix. The columns of **Q**_**r**_ and the zero vector forms the student matrix denoted by **Q**_**s**_ in which the columns forms the universal set of attribute patterns. If *K* attributes are independent, **A** is a zero matrix, **R** with *K* columns is an identity matrix, **Q**_**r**_ with 2^*K*^ − 1 columns does not include the zero vector, and **Q**_**s**_ with 2^*K*^ columns contains all possible combinations of attribute patterns.

We assume that the cognitive requirement for the multiple skills within an item is conjunctive (Maris, 1999), that is, answering an item correctly requires mastery of all the skills required by that item. For the conjunctive model, Example 1 will show the relationship of latent responses on items with q-vectors corresponding to **R** and **Q**_**r**_.

*Example 1 for an independent structure*. Let *K* = 2, **R** = [**r**_**1**_ **r**_**2**_] = $\left[\begin{array}{cc} 1 & 0\\ 0 & 1 \end{array}\right]$, **Q**_**r**_ = [**q**_**1**_ **q**_**2**_ **q**_**3**_] = $\left[\begin{array}{ccc} 1 & 0 & 1\\ 0 & 1 & 1 \end{array}\right]$, and **Q**_**s**_ = [**α**_**1**_ **α**_**2**_ **α**_**3**_ **α**_**4**_] = $\left[\begin{array}{cccc} 0 & 1 & 0 & 1\\ 0 & 0 & 1 & 1 \end{array}\right]$.

Given **Q**_**s**_ and a test Q-matrix of **Q**_**r**_, a latent response matrix **B** = [**η**_**1**_ **η**_**2**_ **η**_**3**_] = $\left[\begin{array}{ccc} 0 & 0 & 0\\ 1 & 0 & 0\\ 0 & 1 & 0\\ 1 & 1 & 1 \end{array}\right]$ can be calculated, in which the entry in row *i* and column *j* is the deterministic latent response of η_*ij*_. If 0 corresponds to F (false) and 1 corresponds to T (true), the logical conjunction and disjunction operators, ∨ and ∧, can be applied to two binary vectors of equal length, by taking the bitwise AND or OR of each pair of bits at corresponding positions. It can be observed that **η**_**3**_ = **η**_**1**_ ∧ **η**_**2**_, where **η**_**3**_ = **η**_**1**_ ∧ **η**_**2**_ is the conjunction of **η**_**1**_ and **η**_2_. This is because the relationship **q**_**3**_ = **q**_1_ ∨ **q**_2_ is true, where **q**_1_ ∨ **q**_2_ is the disjunction of **q**_1_ and **q**_2_.

Example 1 illustrates the following fact. For the conjunctive model, consider two latent response matrices denoted by **B**_1_ and **B**_2_ from two tests corresponding two Q-matrices **Q**_**r**_ and **R**, where denoted as a reachability matrix. It means that **B**_1_ and **B**_2_ can be generated, respectively from the reduced Q-matrix and the reachability matrix based on the universal set of attribute patterns. From the example above, then any columns of the **B**_1_ can be expressed by the columns of the **B**_2_ under the logical AND operation. This is because the augmented algorithm proposed by Ding et al. (2008, 2009) in the generalized Q-matrix theory (Ding et al., 2015) provided the useful fact that any columns of the reduced Q-matrix can be expressed by the columns of the reachability matrix under the logical OR operation. The argument in Example 1 can be adapted to prove the following theorem.

*Theorem 1*. For the conjunctive model, if *K* attributes are independent, then **q**_**j**_ = ∨_*l*∈_*S*__*j*__**r**_**l**_ if and only if η_*ij*_ = ∧_*l*∈_*S*__*j*__η_*il*_, where **α**_*i*_ is any column of **Q**_**s**_ and *S*_*j*_ is a subset of {1, 2, …, *K*}.

*Proof* : If **q**_**j**_ = ∨_*l*∈_*S*__*j*__**r**_**l**_, we need to consider two cases, when η_*ij*_ = 1 and η_*ij*_ = 0. If η_*ij*_ = 1 for **α**_*i*_ as a column of **Q**_**s**_, we know that α_*ki*_ = 1 for all attributes *k* with *q*_*kj*_ = 1 by the definition of the deterministic latent response. That is, examinee *i* has mastered all the skills required by item *j*. Since **q**_**j**_ = ∨_*l*∈_*S*__*j*__**r**_**l**_, then by the definition of conjunction, we can conclude that α_*ki*_ = 1 for all attributes *k* with *r*_*kl*_ = 1 for all *l* ∈ *S*_*j*_. We now use the definition of the deterministic latent response to conclude that η_*il*_ = 1 for all *l* ∈ *S*_*j*_, that is, ∧_*l*∈_*S*__*j*__η_*il*_ = 1. This shows that η_*ij*_ = ∧_*l*∈_*S*__*j*__η_*il*_ when η_*ij*_ = 1. If η_*ij*_ = 0 for **α**_*i*_ as a column of **Q**_**s**_, we know that α_*ki*_ = 0 for at least one of attributes with *q*_*kj*_ = 1 by the definition of the deterministic latent response. That is, examinee *i* has not mastered all the skills required by item *j*. Since *q*_*kj*_ = 1 and **q**_**j**_ = ∨_*l*∈_*S*__*j*__**r**_**l**_, there is an item *l* in *S*_*j*_ such that *r*_*kl*_ = 1. This means that item *l* measured attribute *k*. Since α_*ki*_ = 0, then by the definition of the deterministic latent response, it follows that η_*il*_ = 0 for at least one of items in *S*_*j*_, that is, ∧_*l*∈_*S*__*j*__η_*il*_ = 0. This show that η_*ij*_ = ∧_*l*∈_*S*__*j*__η_*il*_ when η_*ij*_ = 0. Next, we try to prove the converse. First suppose that there exists an attribute *k* ∈ {1, 2, …, *K*} such that ∨_*l*∈_*S*__*j*__*r*_*kl*_ = 1 and *q*_*kj*_ = 0. Since ∨_*l*∈_*S*__*j*__*r*_*kl*_ = 1, we know that there exists an item *l* ∈ *S*_*j*_ with *r*_*kl*_ = 1. Due to the arbitrariness of **α**_*i*_, let **α**_*i*_ = **1** − **e**_*k*_, where **1** = (1 1 … 1)^*T*^ and **e**_*k*_ is the vector with a 1 in the *k*th entry and 0's elsewhere. This is a contradiction, because we know that η_*ij*_ = 1, while ∧_*l*∈_*S*__*j*__η_*il*_ = 0. Similarly, we assume that there exists an attribute *k* ∈ {1, 2, …, *K*} such that ∨_*l*∈_*S*__*j*__*r*_*kl*_ = 0 and *q*_*kj*_ = 1. One can still take **α**_*i*_ = **1** − **e**_*k*_. This is also a contradiction, because we know that η_*ij*_ = 0, while ∧_*l*∈_*S*__*j*__η_*il*_ = 1. The proof is complete.

The important fact about Theorem 1 is that if a latent response matrix is calculated from a Q-matrix, the relationship between the columns in the Q-matrix can be constructed from the relationship between the corresponding columns in the latent response matrix. It should be noted that an observed item response is a function of an underlying latent response and slip and guessing parameters. In other words, the noise introduced in the process is due to slip and guessing parameters.

Next, we will introduce a semi-supervised learning method for Q-matrix specification for the conjunctive model by using the result of Theorem 1 and considering the noise in item responses. Without loss of generality, we begin by arbitrarily assigning q-vector **q**_**j**_ to item *j*. Given a test Q-matrix, written as **Q**_*t*_ = [**R**_*K* × *K*_ **q**_*j*_] = [**r**_**1**_ **r**_**2**_ … **r**_**K**_ **q**_**j**_], where **R** is a reachability matrix specified by subject matter experts and the remaining **q**_**j**_ is unknown. Let **U** = [**X**_*N* × *K*_**Y**_*N* × 1_] be an item response matrix on **Q**_*t*_, where *N* is the sample size. The estimate of **q**_**j**_ can be written as

(3) $$\begin{array}{l} {\hat{\text{q}}}_{j}=\vee_{r_{k}\in {\hat{S}}_{j}}{\text{r}}_{k}, \end{array}$$

where logical OR is applied to the corresponding entries of the columns in the following set of *Ŝ*_*j*_

(4) $$\begin{array}{l} {\hat{S}}_{j}=\underset{S\in \text{P}\left(\left\{r_{1},r_{2},\ldots ,r_{K}\right\}\right)-\emptyset }{\arg\min}{\left(Y_{j}-\wedge_{r_{k}\in S}X_{k}\right)}^{T}\left(Y_{j}-\wedge_{r_{k}\in S}X_{k}\right), \end{array}$$

where P({*r*_1_, *r*_2_, …, *r*_*K*_}) is the power set of the set {**r**_**1**_, **r**_2_, …, **r**_*K*_}. The exhaustive method with time complexity *O*(2^*K*^) provided a simple way to find a global solution of *Ŝ*_*j*_.

#### A Semi-supervised Learning Approach for the Disjunctive Model

For the disjunctive model, the deterministic latent response on an item is correct if and only if an examinee has mastered at least one of the skills required by the item. This is illustrated in Example 2. Similar to what we did in Example 1, Example 2 will show the relationship of latent responses on items with q-vectors corresponding to **R** and **Q**_**r**_.

*Example 2 for an independent structure*. Let *K* = 2, **R** = [**r**_**1**_ **r**_**2**_] = $\left[\begin{array}{cc} 1 & 0\\ 0 & 1 \end{array}\right]$, **Q**_**s**_ = [**α**_**1**_ **α**_**2**_ **α**_**3**_ **α**_**4**_] = $\left[\begin{array}{cccc} 0 & 1 & 0 & 1\\ 0 & 0 & 1 & 1 \end{array}\right]$, and **Q**_**r**_ = [**q**_**1**_ **q**_**2**_ **q**_**3**_] = $\left[\begin{array}{ccc} 1 & 0 & 1\\ 0 & 1 & 1 \end{array}\right]$. From **Q**_**s**_ and **Q**_**r**_, a latent response matrix **W**_1_ = [**w**_**1**_ **w**_**2**_ **w**_**3**_] = $\left[\begin{array}{ccc} 0 & 0 & 0\\ 1 & 0 & 0\\ 0 & 1 & 0\\ 1 & 1 & 1 \end{array}\right]$ can be calculated, in which the entry in row *i* and column *j* is the deterministic latent response of *w*_*ij*_. It can be observed that **w**_**3**_ = **w**_**1**_ ∨ **w**_**2**_. This is because the relationship **q**_**3**_ = **q**_1_ ∨ **q**_2_ is true.

Consider a latent response matrix, denoted by **W**_2_ = [**w**_**1**_ **w**_2_], corresponding to the **R** matrix. The fact illustrated in Example 2 is that any columns of the **W**_1_ can be expressed by the columns of the **W**_2_ under the logical OR operation for the disjunctive model. This is also because the augmented algorithm proposed by Ding et al. (2008, 2009) in the generalized Q-matrix theory (Ding et al., 2015) provided the useful fact that any columns of the reduced Q-matrix can be expressed by the columns of the reachability matrix under the logical OR operation. The following theorem gives the precise statement.

*Theorem 2*. For the disjunctive model, if *K* attributes are independent, then **q**_**j**_ = ∨_*l*∈_*S*__*j*__**r**_**l**_ if and only if *w*_*ij*_ = ∨_*l*∈_*S*__*j*__*w*_*il*_, where **α**_*i*_ is any column of **Q**_**s**_ and *S*_*j*_ is a subset of {1, 2, …, *K*}.

*Proof:* If **q**_**j**_ = ∨_*l*∈_*S*__*j*__**r**_**l**_, we need to consider two cases, when *w*_*ij*_ = 1 and *w*_*ij*_ = 0. If *w*_*ij*_ = 1 for **α**_*i*_ as a column of **Q**_**s**_, we know that α_*ki*_ = 1 for at least one of attributes *k* with *q*_*kj*_ = 1 by the definition of the deterministic latent response. That is, examinee *i* has mastered at least one of the attributes required by item *j*. Without loss of generality, we assume α_*ki*_ = 1 and *q*_*kj*_ = 1. Since **q**_**j**_ = ∨_*l*∈_*S*__*j*__**r**_**l**_, then by the definition of disjunction, we can conclude that *r*_*kl*_ = 1 is true for at least one of *l* ∈ *S*_*j*_. From the definition of the deterministic latent response, it follows that there is at least one item *l* ∈ *S*_*j*_ such that *w*_*il*_ = 1, that is, ∨_*l*∈_*S*__*j*__*w*_*il*_ = 1. This show that *w*_*ij*_ = ∨_*l*∈_*S*__*j*__*w*_*il*_ when *w*_*ij*_ = 1. If *w*_*ij*_ = 0 for **α**_*i*_ as a column of **Q**_**s**_, we know that *w*_*ki*_ = 0 for all of attributes with *q*_*kj*_ = 1 by the definition of the deterministic latent response. That is, examinee *i* has not mastered any skills required by item *j*. Since **q**_**j**_ = ∨_*l*∈_*S*__*j*__**r**_**l**_, examinee *i* has not mastered any skills required by any item *l* ∈ *S*_*j*_. If we suppose that examinee *i* has mastered at least one of attributes required by an item *l* ∈ *S*_*j*_, then *w*_*ij*_ = 1, which is a contradiction. It means that item *l* measured attribute *k*. It follows that *w*_*il*_ = 0 for all of items in *S*_*j*_, that is, ∨_*l*∈_*S*__*j*__*w*_*il*_ = 0, directly from the definition of the deterministic latent response. This show that *w*_*ij*_ = ∨_*l*∈_*S*__*j*__*w*_*il*_ when *w*_*ij*_ = 0. Next, we use a proof by contradiction to prove the converse. First assume that there exists an attribute *k* ∈ {1, 2, …, *K*} such that ∨_*l*∈_*S*__*j*__*r*_*kl*_ = 1 and *q*_*kj*_ = 0. Since ∨_*l*∈_*S*__*j*__*r*_*kl*_ = 1, we know that there exists an item *l* ∈ *S*_*j*_ with *r*_*kl*_ = 1. Due to the arbitrariness of **α**_*i*_, let **α**_*i*_ = **e**_*k*_, where **e**_*k*_ is the vector with a 1 in the *k*th entry and 0's elsewhere. Then, we have*w*_*il*_ = 1 and *w*_*ij*_ = 0. Since*w*_*ij*_ = ∨_*l*∈_*S*__*j*__*w*_*il*_, we know that *w*_*ij*_ = 1 and arrive at a contradiction. Similarly, we assume that there exists an attribute *k* ∈ {1, 2, …, *K*} such that ∨_*l*∈_*S*__*j*__*r*_*kl*_ = 0 and *q*_*kj*_ = 1. One can still take **α**_*i*_ = **e**_*k*_. This is also a contradiction, because we know that *w*_*ij*_ = 1, while ∧_*l*∈_*S*__*j*__*w*_*il*_ = 0. The proof is complete.

The important fact about Theorem 2 is that one can derive the relationship between the columns of a Q-matrix from the relationship between the columns of corresponding latent response matrix. For considering the noise introduced in item responses due to slipping and guessing, we will introduce a semi-supervised learning method for Q-matrix specification for the disjunctive model by using the result of Theorem 2. Without loss of generality, we begin by arbitrarily assigning a q-vector to **q**_**j**_. Given a test Q-matrix, written as **Q**_*t*_ = [**R**_*K* × *K*_ **q**_*j*_] = [**r**_**1**_ **r**_**2**_ … **r**_**K**_ **q**_**j**_], where **R** is a reachability matrix specified by subject matter experts and the remaining **q**_**j**_ is unknown. Let **U** = [**X**_*N* × *K*_ **Y**_*N* × 1_] be an item response matrix on **Q**_*t*_. The estimate of **q**_**j**_ can be written as

(5) $$\begin{array}{l} {\hat{\text{q}}}_{j}=\vee_{r_{k}\in {\hat{S}}_{j}}{\text{r}}_{k}, \end{array}$$

where logical OR is applied to the corresponding entries of the columns in the following set of *Ŝ*_*j*_

(6) $$\begin{array}{l} {\hat{S}}_{j}=\underset{S\in \text{P}\left(\left\{r_{1},r_{2},\ldots ,r_{K}\right\}\right)-\emptyset }{\arg\min}{\left(Y_{j}-\vee_{r_{k}\in S}X_{k}\right)}^{T}\left(Y_{j}-\vee_{r_{k}\in S}X_{k}\right), \end{array}$$

where P({*r*_1_, *r*_2_, …, *r*_*K*_}) is the power set of the set {**r**_**1**_, **r**_2_, …, **r**_*K*_}. The exhaustive method with time complexity *O*(2^*K*^) provided a simple way to find a global solution of *Ŝ*_*j*_.

## A Simulation Study

### Study Design

A simulation study was conducted to investigate the performance of the new method under five factors, such as sample size, item parameters for items corresponding to a reachability matrix, item parameters for new or raw items with unknown q-vectors, two cognitive diagnostic models (the DINA and DINO model), and two designs. Five attributes were considered in the simulation study. Matlab 2015a and R-3.6.1 were used for estimating unknown Q-matrix and analyzing real data below.

In the simulation study, a test Q-matrix **Q**_**t**_ = [**R** **Q**_**r**_] consists of an identity or a reachability matrix and a reduced Q-matrix, where the reduced Q-matrix with 31 items includes all non-zero possible q-vectors. The number of examinees has 10 levels, such as *N*=30, 60, …, and 300. Item parameters for **R** and **Q**_**r**_ have 10 levels, such as 0, 0.05, …, and 0.45. In general, for the DINA or DINO model, a high quality or “good” item will have small slip and guessing parameters (Rupp et al., 2010), which means that the noise are small.

Random and optimal designs were considered in the simulation study. For the random design, attribute patterns for examinees were generated by taking each of the 2^5^ possible patterns with equal probability for each sample size. From the proof of Theorem 1 above, we know that the following set of attribute patterns for examinees plays a very important role in discriminating latent response vectors of different q-vectors under the DINA model

(7) $$\begin{array}{l} S_{DINA}=\left\{1-{\text{e}}_{1}1-{\text{e}}_{2},\ldots ,1-{\text{e}}_{K}\right\}\left\{\left[\begin{array}{c} 0\\ 1\\ \vdots \\ 1 \end{array}\right],\left[\begin{array}{c} 1\\ 0\\ \vdots \\ 1 \end{array}\right],\ldots ,\left[\begin{array}{c} 1\\ 1\\ \vdots \\ 0 \end{array}\right]\right\} \end{array}$$

where **e**_*k*_ is the vector with a 1 in the *k*th entry and 0's otherwise. From the proof of Theorem 2 above, another set of attribute patterns for examinees plays a very important role in discriminating latent response vectors of different q-vectors under the DINO model as follows

(8) $$\begin{array}{l} S_{DINO}=\left\{{\text{e}}_{1},{\text{e}}_{2},\ldots ,{\text{e}}_{K}\right\}\left\{\left[\begin{array}{c} 1\\ 0\\ \vdots \\ 0 \end{array}\right],\left[\begin{array}{c} 0\\ 1\\ \vdots \\ 0 \end{array}\right],\ldots ,\left[\begin{array}{c} 0\\ 0\\ \vdots \\ 1 \end{array}\right]\right\}, \end{array}$$

where **e**_*k*_ is the vector with a 1 in the *k*th entry and 0's otherwise. For the optimal design, attribute patterns for examinees under the DINA or DINA model were randomly drawn with replacement from the set of *S*_*DINA*_ or *S*_*DINO*_, respectively. Optimal designs for two models are possible to meet the needs of learners at different stages of skills and knowledge acquisition. For example, the attribute patterns in *S*_*DINO*_ containing only one skill. This condition is really improbable for summary assessments in real situations, but is expected to be common for novice learners with respect to the new content to be learned in formative assessments or classroom assessments.

### Data Simulation

Simulated data were generated using five attributes. Based on the simulated Q-matrix, item parameters, and attribute patterns, item responses are generated in the following way

(9) $$\begin{array}{l} X_{ij}=\left\{ \begin{array}{cc} 1, & if\;u\leq P_{j}\left(\alpha_{i}\right),\\ 0, & otherwise, \end{array}\right. \end{array}$$

where *u* is a random value from a Uniform (0, 1) distribution and *P*_*j*_(**α**_**i**_) is the item response function of the DINA or DINO model. A total of 4,000 conditions were simulated (10 sample sizes × 10 item parameters × 10 item parameters × 2 models × 2 designs). Thirty replication data sets were simulated for each condition.

### Evaluation Criterion

The performance of the new method is evaluated in terms of the correct recovery rate (CRR) of q-entries. The correct recovery rate equals the ratio of the number of correct q-entries in the estimated Q-matrix to the total number of q-entries (Chiu, 2013)

(10) $$\begin{array}{l} \text{CRR}=\frac{1}{KM}\overset{K}{\underset{k=1}{\sum }}\overset{M}{\underset{j=1}{\sum }}\text{I}\left({\hat{q}}_{kj}=q_{kj}\right), \end{array}$$

where *M* = 31 is the number of columns of the unknown Q-matrix **Q**_**r**_, *q*_*kj*_ is an (*k, j*)th entry of the simulated **Q**_**r**_, and ${\hat{q}}_{kj}$ is an (*k, j*) entry of the ${\hat{\text{Q}}}_{\text{r}}$ estimated from the new method. The mean and standard deviation of the CRR values of the 30 replications were reported for each condition.

### Results

Table 1 lists descriptive statistics of correct recovery rate of q-entries for two models and two designs across other conditions. It is clear that the mean of correct recovery rates of q-entries tends to increase as sample size increases, but sample size has slightly affected the standard deviations of correct recovery rates. It should be noted that the mean of correct recovery rates of the optimal design is larger than that of the random design. The semi-supervised learning method for q-matrix specification performed similarly under two cognitive diagnostic models. In addition, since there are 32 possible attribute patterns, no all attribute patterns can be observed in the first sample size condition (*N* = 30). This might lead to lower rate of correct recovery observed for this condition.

**Table 1:** Mean and standard deviation (in brackets) of correct recovery rate of q-entries for two models and two designs.

**Sample size**	**The DINA model**		**The DINO model**	
	**Random design**	**Optimal design**	**Random design**	**Optimal design**
30	0.651 (0.126)	0.720 (0.147)	0.653 (0.126)	0.721 (0.146)
60	0.699 (0.145)	0.769 (0.153)	0.700 (0.144)	0.770 (0.151)
90	0.725 (0.149)	0.796 (0.152)	0.726 (0.149)	0.796 (0.151)
120	0.742 (0.151)	0.815 (0.149)	0.743 (0.151)	0.815 (0.148)
150	0.756 (0.153)	0.827 (0.146)	0.754 (0.153)	0.829 (0.145)
180	0.764 (0.153)	0.839 (0.143)	0.765 (0.153)	0.838 (0.144)
210	0.772 (0.153)	0.847 (0.141)	0.772 (0.151)	0.846 (0.141)
240	0.779 (0.152)	0.854 (0.138)	0.777 (0.152)	0.854 (0.138)
270	0.784 (0.151)	0.858 (0.137)	0.783 (0.152)	0.858 (0.137)
300	0.789 (0.151)	0.863 (0.135)	0.789 (0.152)	0.864 (0.135)
Mean	0.746 (0.154)	0.819 (0.151)	0.746 (0.154)	0.819 (0.150)

Table 2 shows the correct recovery rates of q-entries from the new method with sample size of 300 for the DINA model under the random design. From correct recovery rates of q-entries, when item parameters for items with known (i.e., the reachability matrix) and unknown q-vectors are ≤ 0.2, most of the average of correct recovery rates of q-entries for the semi-supervised method are larger than or equal to 0.9. From trends of marginal means of last rows and columns in Table 2, item parameters of the reachability matrix have a relatively larger impact on the performance of the semi-supervised method than item parameters with unknown q-vectors.

**Table 2:** The correct recovery rates of q-entries with sample size of 300 for the DINA model and random design.

**Item parameters for the R**	**Item parameters for the new items**										
	**0.00**	**0.05**	**0.10**	**0.15**	**0.20**	**0.25**	**0.30**	**0.35**	**0.40**	**0.45**	**M**
0.00	**1.000**	**1.000**	**1.000**	**0.999**	**0.998**	**0.993**	**0.974**	**0.929**	0.837	0.665	0.939
0.05	**1.000**	**1.000**	**0.997**	**0.995**	**0.991**	**0.976**	**0.946**	**0.908**	0.791	0.642	0.925
0.10	**0.994**	**0.992**	**0.984**	**0.984**	**0.967**	**0.954**	**0.927**	0.857	0.767	0.632	0.906
0.15	**0.974**	**0.968**	**0.958**	**0.954**	**0.932**	**0.915**	0.866	0.807	0.724	0.608	0.870
0.20	**0.927**	**0.922**	**0.910**	**0.901**	0.881	0.860	0.826	0.776	0.692	0.591	0.829
0.25	0.866	0.846	0.850	0.839	0.825	0.802	0.776	0.733	0.652	0.567	0.775
0.30	0.791	0.801	0.782	0.793	0.760	0.735	0.727	0.670	0.624	0.563	0.725
0.35	0.728	0.718	0.720	0.709	0.709	0.698	0.683	0.637	0.604	0.546	0.675
0.40	0.673	0.686	0.681	0.680	0.668	0.643	0.620	0.608	0.589	0.527	0.638
0.45	0.647	0.634	0.623	0.620	0.615	0.612	0.604	0.575	0.575	0.537	0.604
M	0.860	0.857	0.851	0.847	0.835	0.819	0.795	0.750	0.686	0.588	0.789

*The bold values are larger than 0.9*.

Table 3 presents the correct recovery rates of q-entries from the new method with sample size of 300 for the DINA model under the optimal design. From correct recovery rates of q-entries, when item parameters for items with known and unknown q-vectors are ≤ 0.25, the average of correct recovery rates of q-entries for the semi-supervised method are larger than or equal to 0.9. However, item parameters for known q-vectors have slightly larger impact on the performance of the semi-supervised method than for unknown q-vectors, because the row means decreased more quickly than the column means. We need to compare the Tables 2, 3 to see which designs are promising. The number of correct recovery rates above 0.9 in Table 3 were found to be larger than that of Table 2. Tables 4, 5 show the correct recovery rates of q-entries from the new method with sample size of 300 for the DINO model under the random and optimal design. It can be observed that results for the DINO model are the same as those for the DINA model described above.

**Table 3:** The correct recovery rates of q-entries with sample size of 300 for the DINA model and optimal design.

**Item parameters for the R**	**Item parameters for the new items**										
	**0.00**	**0.05**	**0.10**	**0.15**	**0.20**	**0.25**	**0.30**	**0.35**	**0.40**	**0.45**	**M**
0.00	**1.000**	**1.000**	**1.000**	**1.000**	**1.000**	**1.000**	**0.999**	**0.991**	**0.939**	0.780	0.971
0.05	**1.000**	**1.000**	**1.000**	**1.000**	**1.000**	**0.999**	**0.997**	**0.981**	**0.917**	0.754	0.965
0.10	**1.000**	**1.000**	**1.000**	**0.999**	**0.998**	**0.995**	**0.981**	**0.955**	0.871	0.714	0.951
0.15	**1.000**	**1.000**	**0.998**	**0.992**	**0.988**	**0.981**	**0.959**	**0.915**	0.841	0.688	0.936
0.20	**0.986**	**0.986**	**0.982**	**0.973**	**0.962**	**0.952**	**0.918**	0.875	0.785	0.661	0.908
0.25	**0.959**	**0.952**	**0.947**	**0.933**	**0.926**	**0.909**	0.879	0.829	0.754	0.624	0.871
0.30	**0.930**	**0.914**	**0.912**	**0.909**	0.883	0.865	0.832	0.786	0.712	0.610	0.835
0.35	0.886	0.888	0.880	0.865	0.847	0.813	0.780	0.724	0.662	0.573	0.792
0.40	0.847	0.834	0.816	0.804	0.774	0.753	0.721	0.679	0.629	0.562	0.742
0.45	0.749	0.738	0.717	0.720	0.688	0.687	0.656	0.619	0.581	0.556	0.671
M	0.936	0.931	0.925	0.920	0.907	0.895	0.872	0.835	0.769	0.652	0.864

*The bold values are larger than 0.9*.

**Table 4:** The correct recovery rates of q-entries with sample size of 300 for the DINO model and random design.

**Item parameters for the R**	**Item parameters for the new items**										
	**0.00**	**0.05**	**0.10**	**0.15**	**0.20**	**0.25**	**0.30**	**0.35**	**0.40**	**0.45**	**M**
0.00	**1.000**	**1.000**	**1.000**	**0.999**	**0.998**	**0.990**	**0.976**	**0.929**	0.840	0.685	0.942
0.05	**1.000**	**0.999**	**0.998**	**0.996**	**0.991**	**0.980**	**0.946**	0.889	0.796	0.650	0.925
0.10	**0.996**	**0.991**	**0.992**	**0.985**	**0.968**	**0.945**	**0.918**	0.848	0.767	0.639	0.905
0.15	**0.971**	**0.969**	**0.963**	**0.947**	**0.931**	**0.912**	0.864	0.806	0.724	0.604	0.869
0.20	**0.927**	**0.921**	**0.915**	**0.905**	0.885	0.854	0.819	0.763	0.687	0.578	0.825
0.25	0.855	0.854	0.865	0.842	0.831	0.797	0.773	0.733	0.665	0.572	0.779
0.30	0.787	0.795	0.789	0.774	0.763	0.743	0.722	0.677	0.629	0.571	0.725
0.35	0.734	0.721	0.717	0.720	0.720	0.686	0.662	0.634	0.597	0.550	0.674
0.40	0.677	0.678	0.689	0.675	0.660	0.654	0.623	0.624	0.573	0.536	0.639
0.45	0.632	0.630	0.628	0.633	0.611	0.608	0.600	0.586	0.571	0.525	0.603
M	0.858	0.856	0.856	0.848	0.836	0.817	0.790	0.749	0.685	0.591	0.789

*The bold values are larger than 0.9*.

**Table 5:** The correct recovery rates of q-entries with sample size of 300 for the DINO model and optimal design.

**Item parameters for the R**	**Item parameters for the new items**										
	**0.00**	**0.05**	**0.10**	**0.15**	**0.20**	**0.25**	**0.30**	**0.35**	**0.40**	**0.45**	**M**
0.00	**1.000**	**1.000**	**1.000**	**1.000**	**1.000**	**1.000**	**0.999**	**0.992**	**0.939**	0.780	0.971
0.05	**1.000**	**1.000**	**1.000**	**1.000**	**1.000**	**1.000**	**0.995**	**0.973**	**0.916**	0.744	0.963
0.10	**1.000**	**1.000**	**1.000**	**1.000**	**0.998**	**0.994**	**0.983**	**0.954**	0.873	0.715	0.952
0.15	**0.998**	**0.999**	**0.996**	**0.995**	**0.985**	**0.978**	**0.957**	**0.922**	0.836	0.695	0.936
0.20	**0.991**	**0.986**	**0.982**	**0.970**	**0.961**	**0.946**	**0.916**	0.873	0.795	0.666	0.909
0.25	**0.957**	**0.951**	**0.951**	**0.935**	**0.925**	**0.909**	0.885	0.829	0.752	0.628	0.872
0.30	**0.922**	**0.910**	**0.913**	**0.902**	0.894	0.866	0.840	0.772	0.705	0.595	0.832
0.35	0.887	0.882	0.870	0.862	0.842	0.807	0.787	0.731	0.678	0.571	0.792
0.40	0.838	0.830	0.812	0.802	0.788	0.749	0.715	0.675	0.620	0.560	0.739
0.45	0.737	0.733	0.733	0.703	0.690	0.663	0.645	0.619	0.595	0.544	0.666
M	0.933	0.929	0.926	0.917	0.908	0.891	0.872	0.834	0.771	0.650	0.863

*The bold values are larger than 0.9*.

## Real Data Analysis

The purpose of the real data analysis is to examinee whether the proposed method is promising for a non-independent structure under the conjunctive model based on an intuitive fact from the following example.

*Example 3 for an unstructured hierarchy under the conjunctive model*. Let *K* = 3, **R** = [**r**_**1**_ **r**_2_ **r**_3_] = $\left[\begin{array}{ccc} 1 & 1 & 1\\ 0 & 1 & 0\\ 0 & 0 & 1 \end{array}\right]$, **Q**_**r**_ = [**q**_**1**_ **q**_2_ **q**_3_ **q**_4_] = $\left[\begin{array}{cccc} 1 & 1 & 1 & 1\\ 0 & 1 & 0 & 1\\ 0 & 0 & 1 & 1 \end{array}\right]$, and **Q**_**s**_ = [**α**_0_ **α**_**1**_ **α**_**2**_ **α**_**3**_ **α**_**4**_] = $\left[\begin{array}{ccccc} 0 & 1 & 1 & 1 & 1\\ 0 & 0 & 1 & 0 & 1\\ 0 & 0 & 0 & 1 & 1 \end{array}\right]$. From the ideal response matrix **B** = [**η**_**1**_ **η**_**2**_ **η**_**3**_ **η**_4_] = $\left[\begin{array}{cccc} 0 & 0 & 0 & 0\\ 1 & 0 & 0 & 0\\ 1 & 1 & 0 & 0\\ 1 & 0 & 1 & 0\\ 1 & 1 & 1 & 1 \end{array}\right]$, it can be observed that **η**_4_ = **η**_2_ ∧ **η**_3_ or **η**_4_ = **η**_1_ ∧ **η**_2_ ∧ **η**_3_. This is because the relationship **q**_4_ = **q**_2_ ∨ **q**_3_ or **q**_4_ = **q**_1_ ∨ **q**_2_ ∨ **q**_3_ is true.

A common data set pertaining to fraction-subtraction data contains 20 items and 536 examines (de la Torre and Douglas, 2004). In our real data analysis, we focused on the analysis of a subset of test items where the expert Q-matrix comes from **Table 7** both in de la Torre (2008) or DeCarlo (2012). The labels given to the five skills are (A1) performing basic fraction-subtraction operation, (A2) simplifying/reducing, (A3) separating whole numbers from fractions, (A4) borrowing one from whole number to fraction, and (A5) converting whole numbers to fractions.

We assumed the corresponding Q-matrix of items 3, 8, 9, 12, and 10 known since these item parameters are relatively small and the q-vectors of other items are combinations of q-vectors for these five items. Then, the semi-supervised method was applied to estimate q-vectors for the other 10 items. Results in Table 6 show that the agreement rate of q-entries between the estimate and expert Q-matrix on the 10 items is 84%. The estimated q-entries suggest that items 4, 7, 13, 14, and 15 do not require attribute 2 (simplifying/reducing). Item 4 (similar to item 14) do not required attribute A2, which is consistent with results from DeCarlo (2012). Items 7, 13, and 15 can be answered correctly by using attributes required by item 12. The estimated q-vector of item 1 has largest discrepancy with the expert q-vector. The reason might be that solving item 1 correctly needs to find a common denominator and then performs basic fraction-subtraction operation. The guessing and slip parameter of item 1 are 0.0001 and 0.2769 under the expert q-vector, respectively. The guessing and slip parameter of item 1 are 0.3408 and 0.0716 under the estimated q-vector, respectively. Since item 1 requires an extra attribute (i.e., find a common denominator), the slip parameter for the expert q-vector is relatively large, while the estimated q-vector contains some unnecessary attributes, the guessing parameter is relatively large. In the estimated Q-matrix, attribute A4 has been added to item 11.The guessing probability of item 11 increased sensibly (from 0.10 to 0.48). It indicated that attribute A4 is not necessary for item 11 because this item is different from items 7, 12, and so on.

**Table 6:** The expert and estimated Q-matrix and item parameters estimates of the DINA model for the fractional subtraction data.

**No**.	**Items**	**The expert Q-matrix and item parameters estimates of the DINA model**							**The estimated Q-matrix and item parameters estimates of the DINA model**						
		**A1**	**A2**	**A3**	**A4**	**A5**	**ĝ**	**ŝ**	**A1**	**A2**	**A3**	**A4**	**A5**	**ĝ**	**ŝ**
1	$\frac{3}{4}-\frac{3}{8}$	1	0	0	0	0	0.00	0.28	1	0	**1**	**1**	**1**	0.34	0.07
2	$3\frac{1}{2}-2\frac{3}{2}$	1	1	1	1	0	0.21	0.12	1	1	1	1	0	0.21	0.11
3	$\frac{6}{7}-\frac{4}{7}$	1	0	0	0	0	0.14	0.04	1	0	0	0	0	0.10	0.05
4	$3-2\frac{1}{5}$	1	1	1	1	1	0.12	0.13	1	**0**	1	1	1	0.12	0.18
5	$3\frac{7}{8}-2$	1	0	1	0	0	0.34	0.25	1	0	1	0	0	0.35	0.25
6	$4\frac{4}{12}-2\frac{7}{12}$	1	1	1	1	0	0.03	0.23	1	1	1	1	0	0.03	0.23
7	$4\frac{1}{3}-2\frac{4}{3}$	1	1	1	1	0	0.07	0.08	1	**0**	1	1	0	0.07	0.08
8	$\frac{11}{8}-\frac{1}{8}$	1	1	0	0	0	0.16	0.05	1	1	0	0	0	0.09	0.04
9	$3\frac{4}{5}-3\frac{2}{5}$	1	0	1	0	0	0.08	0.06	1	0	1	0	0	0.04	0.04
10	$2-\frac{1}{3}$	1	0	1	1	1	0.17	0.07	1	0	1	1	1	0.15	0.09
11	$4\frac{5}{7}-1\frac{4}{7}$	1	0	1	0	0	0.10	0.10	1	0	1	**1**	0	0.48	0.07
12	$7\frac{3}{5}-\frac{4}{5}$	1	0	1	1	0	0.03	0.13	1	0	1	1	0	0.05	0.14
13	$4\frac{1}{10}-2\frac{8}{10}$	1	1	1	1	0	0.13	0.16	1	**0**	1	1	0	0.13	0.16
14	$4-1\frac{4}{3}$	1	1	1	1	1	0.02	0.20	1	**0**	1	1	1	0.01	0.24
15	$4\frac{1}{3}-1\frac{5}{3}$	1	1	1	1	0	0.01	0.18	1	**0**	1	1	0	0.01	0.19

*The bold values are the changes*.

The generalized DINA model (GDINA; de la Torre, 2011), the DINA model, the linear logistic model (LLM; Fischer, 1995), and the reduced reparametrized unified model (R-RUM; Hartz, 2002) were applied to fit the fraction-subtraction data with the expert or estimated Q-matrix. Under the DINA model, the means of the estimates of the guessing and slip parameter for the expert Q-matrix are 0.1080 and 0.1381, respectively, while for the revised Q-matrix, they are 0.1440 and 0.1295, respectively. It means that the estimates of the slip parameter become lower, but the guessing parameters tend to be larger. Table 7 presents fit results for the fraction subtraction data using the expert and estimated q-matrix. The LLM with the estimated Q-matrix is the best-fitting CDM and the R-RUM with the estimated Q-matrix is slightly worse, whereas the estimated Q-matrix performed worse than the expert Q-matrix only in the DINA model.

**Table 7:** Fit results for the fraction subtraction data using the expert and estimated Q-matrix.

**Q-matrix**	**CDM**	**–LL2**	**AIC**	**BIC**
Expert Q-matrix	GDINA	6,695	7,133	8,071
Estimated Q-matrix	GDINA	6,548	6,910	7,686
Expert Q-matrix	DINA	6,912	7,034	7,295
Estimated Q-matrix	DINA	7,030	7,152	7,413
Expert Q-matrix	LLM	6,595	6,781	7,179
Estimated Q-matrix	LLM	6,523	6,707	7,102
Expert Q-matrix	R-RUM	6,696	6,882	7,280
Estimated Q-matrix	R-RUM	6,543	6,727	7,122

*−2LL, −2 log likelihood; AIC, Akaike's information criterion; BIC, Bayesian information criterion (Chen et al., 2013)*.

## Conclusion and Discussion

The supervised methods rely on a provisional Q-matrix for a whole test, the estimates of examinees' attribute patterns and their accuracy. It is not suitable for the case of a provisional Q-matrix with a large amount of misspecification. The purpose of this study is to propose the semi-supervised method under independent structure based on item responses and a reachability R matrix corresponding to a small part of test item specified by subject matter experts. The new method doesn't need to estimate examinees' attribute patterns. The main conclusion of this study is that the new method will play a very important role in assist subject matter experts for Q-matrix specification because it is hard to correctly specify a Q-matrix with a large number of test items by subject matter experts. It may be useful for cognitive diagnostic assessment to facilitate teaching and learning.

The generalized Q-matrix theory has been shown that each column in the reduced Q-matrix can be expressed as a logical disjunction of some of columns of the reachability matrix. With the aid of this theory, this study takes a look inside a latent response matrix and reveals an interesting and useful relationship hidden in its columns. If a latent response matrix is calculated from a Q-matrix under the conjunctive model, a column in the latent response matrix is the conjunction of some other columns in this matrix if and only if the corresponding column of the Q-matrix can be written as the disjunction of their corresponding columns. While for the disjunctive model, the columns of the latent response matrix have exactly the same disjunction relationships as the columns of the Q-matrix. Because any conjunction or disjunction relationship among the columns of a latent response matrix would imply a disjunction relationship among the columns of a Q-matrix, then we are expected that the relationship between the columns in the Q-matrix can be constructed from the relationship between the corresponding columns in an observed response matrix, resulting from the latent response matrix by adding the noise or random errors. Another reason for this expectation is that each entry in the observed response matrix is modeled as a noisy observation of the corresponding entry in the latent response matrix through slip and guessing parameters (Junker and Sijtsma, 2001) and the discrepancies between the latent and observed response matrices are considered as random errors (Tatsuoka, 1987).

From the key theoretical results above, the semi-supervised method and an optimal design were then proposed for Q-matrix specification based on test response data and a reachability matrix specified by subject matter experts, and the simulation study was conducted to investigate the performance of the new method and the optimal design for examinee sampling in terms of the CRR of q-entries. From the CRR of q-entries, it is clear found that: (a) for the random design, when item parameters for items with known and unknown q-vectors are ≤ 0.20, the average of CRRs of q-entries for the semi-supervised method is larger than or equal to 0.9, (b) for the optimal design, when item parameters for items with known and unknown q-vectors are ≤ 0.25, the average of CRRs of q-entries for the semi-supervised method is larger than or equal to 0.9, and (c) item parameters of the reachability matrix have a larger impact on the performance of the semi-supervised method than item parameters with unknown q-vectors.

Finally, based on the results obtained in this study, some problems worthy of study in the future are put forward. First, how to effectively use the most of data or information on some other items for which experts have also specified q-vectors, because as the increase of the number of item specified q-vectors, the time complexity (more specifically, exponential time) of the exhaustive method grows much faster? If the number of items is increased to double or triple the number of attributes corresponding to the reachability matrix, one should investigate whether choosing a small part of items with high quality will reduce the noise of the responses and improve the estimation of q entries of unknown items. Second, in the simulation study, we know exactly how many attributes all items include. However, in the real situation, some items with unknown Q-matrix may mix additional attributes not specified in the reachability matrix because we haven't reviewed all items. Thus, we should explore a novel or revised method for identifying the possibility of extra attribute(s). Third, if the Q-matrix obtained from the semi-supervised method is taken as an initial matrix or a provisional Q-matrix of the existing supervised methods, is it possible to further improve the recovery of Q-matrix? From the results of the study, it can be seen that item parameters or random errors of item responses have an impact on the recovery of Q-matrix. If there is a method to reduce noise in item responses, the recovery of Q-matrix may be further improved. We only considered the small set of items with known q-vectors and fixed item parameters. Additional work is needed to further examine the impact of not only error patterns for known q-vectors but different item parameters for test items. Fourth, the current study focused on the DINA and DINO model only. In the future, the proposed method should be applied to general families of cognitive diagnostic models such as the generalized DINA model (de la Torre, 2011), the log-linear cognitive diagnostic model (Henson et al., 2009), the general diagnostic model (von Davier, 2008), testlet cognitive diagnosis model (Zhan et al., 2018), or polytomous cognitive diagnosis models (Chen and de la Torre, 2018; Ma, 2019). Lastly, since only the independent attribute structure in the simulation study and hierarchy structures for the conjunctive model in real data analysis were considered, the proposed method for other attribute hierarchies with different cognitive assumptions is worth studying.

## Data Availability Statement

Publicly available datasets were analyzed in this study. This data can be found here: https://www.rdocumentation.org/packages/CDM/versions/7.4-19/topics/fraction.subtraction.data.

## Author's Note

Based on the fact that any columns of a reduced Q-matrix can be expressed by the columns of a reachability R matrix under the logical OR operation, a semi-supervised learning approach and an optimal design for examinee sampling were proposed for Q-matrix specification under the conjunctive and disjunctive model. This method only required subject matter experts specifying a R matrix corresponding to a small part of test items. Simulation and real data analysis showed that the new method with the optimal design is promising in terms of correct recovery rates of q-entries.

## Author Contributions

WW, LS, and TW conducted a design of the study, data analysis, paper writing, and revision. SD revised the paper. PG and JX give some descriptions of data analysis. All authors contributed to the article and approved the submitted version.

## Conflict of Interest

The authors declare that the research was conducted in the absence of any commercial or financial relationships that could be construed as a potential conflict of interest.
